# Couplet Analysis of Linguistic Topology Using Deep Neural Networks in Cognitive Linguistics

**DOI:** 10.1155/2022/9123922

**Published:** 2022-10-11

**Authors:** Dongmei Zhu, Nan Wang, Fuqiang Yang

**Affiliations:** ^1^School of Foreign Languages, Henan University, Kaifeng 475001, China; ^2^College of Literature and Art, Shihezi University, Shihezi 832000, China; ^3^School of Electrical & Information Engineering, Kunsan National University, Gunsan, Jeollabuk-do 54150, Republic of Korea

## Abstract

The work reported here primarily aims to realize the automatic generation of couplets using the linguistic topology of deep neural network (DNN). First, the symmetry, topology, and cognitive linguistics of language are explored to lay a theoretical foundation for subsequent model establishment and analysis. Then, the recurrent neural network (RNN) is employed to build the Seq2Seq model, and Liweng's Guide to Rhyme (an ancient Chinese enlightenment reading material to poetry creation) is imported into the Seq2Seq model as a basic corpus. Eventually, the entire system is implemented automatically on TensorFlow. The system undergoes tests of the five-character quatrain, the seven-character quatrain, the couplet, and the part-of-speech detection. Results demonstrate that both the first and the second lines of the couplet present an excellent correspondence regarding sentences and words. After some famous verses are entered, the second line of the couplet obtained is quite vivid and appropriate. Meanwhile, the results can be generated quickly and meet the requirements on rhyme and couplet matching. This model can input verses according to users' own needs and generate the second line of the couplet quickly, showing good correspondence in words, part-of-speech, and sentence pattern. Because the couplet belongs to Chinese traditional culture, it has a strong local Chinese cultural flavor. The system designed based on computer technology can help people learn and experience the charm of couplets.

## 1. Introduction

In the early 20th century, the computer was just born, while scientists had already begun to use computers to analyze human language. The main purpose at that time was for strategic needs because it was in the age of war, and the decryption and transmission of information belonged to high-level secrets. Later, when it came to the Cold War Period, the United States used the language analysis function of computers to decipher Russian confidential information into English and finally achieved real-time monitoring of Soviet defense scientific and technological information [1]. In this process, the US research team got inspiration from the information deciphering process of the Soviet Union; they believed that they only used different coding expressions for the same semantics while deciphering different languages. This inspiration fulfilled the early machine translation idea and also created a new era of natural language [2].

From 1970 to 1980, because the international environment entered a period of slightly stable relaxation, a series of science and technology developed rapidly. Computer was one of the important technologies, and simultaneously, Udine's million-level corpus was processed. An independent field with natural language processing as the core was established in the general direction of artificial intelligence in the following decade [3]. At this time, two different branches emerged in natural language processing: the rule school and the statistics school. The branch based on linguistic theories was the rule school. After linguistic scientists explained language phenomena, scholars of this school described the language using explanations made by linguistics. The other school was based on data statistics of corpus. This school focused on mathematics and could find laws from large numbers of language materials to represent natural language. Until around 1990, natural language processing greatly affected people's lives with the continuous development of Internet technology. In China, people can use Chinese search engines to edit Chinese words. Many problems have been solved with a series of breakthroughs of algorithms. These algorithms are summarized as “machine learning” [4]. These algorithms are basically based on the working principles of the brain and neurons and gradually mature by simulating some human cognition and behaviors. Because many algorithms in machine learning can efficiently handle multi-dimensional and nonlinear problems, they have attracted many research teams.

Comerica used simple complex topological structures to simulate political and cultural structures. They translated wedge, cone, and hanging operations into the language of political structures and showed how these structures correspond to merged structures and the introduction of intermediaries. They introduced the concepts of agent's viability and political system stability, examined their interaction with simple and complex topologies, and expressed the interaction between them in the language of topology theory as much as possible [5]. Kiran et al. proposed a framework for language restoration, which included a network-based view of the brain regions involved in language restoration. Besides, the mechanism model of language recovery was established to explain individual differences in behavior, network topology, and treatment response [6]. Shen and Ho combined information and communication technology to improve teaching and learning. Through latent semantic analysis of the topic, they summarized the state of knowledge accumulation and helped people quickly understand the articles [7].

This couplet analysis of language topology based on deep neural network (DNN) first discusses the symmetry and topology of language and then establishes the Seq2Seq model through the recurrent neural network (RNN). Besides, a corpus is imported into the Seq2Seq model. Finally, the TensorFlow framework is used to realize the automatic operation of the whole system. It is hoped to establish and match couplets quickly and efficiently through deep learning. The innovation lies in the optimization of the artificial neural network (ANN) and using the RNN to achieve the repeated transmission of text content, which minimizes the final matching between the second line of a couplet and the first line of a couplet.

## 2. Theoretical Analysis and Research Design

### 2.1. Symmetry of Language

To some extent, language signs can be regarded as symmetric operation signs. These signs can be combined through symmetric conversion to output the language. During language expression, each character is a conversion operation. The symmetry of language is an attribute of all languages [8, 9]. Symmetry is one of the inherent properties of language. Chinese is precisely one of the best languages in the world. Because Chinese is gradually transformed from hieroglyphics, the symmetry shown in Chinese is even more unique than other languages. Chinese has perfect symmetry in text, phonetics, grammar, and rhetoric, which is also a big difference from other languages [10]. The overall shape of Chinese characters is square, and each word has a special meaning and a single syllable, which once again highlights the superiority of Chinese.

Regarding dimensional analysis, Chinese sentences are more prominent in terms of symmetry. There are many representative Chinese writing styles, such as poetry and couplet, which are similar to parallel prose and antithetical sentences in ancient Chinese literature [11]. In the earlier Shang Dynasty (1600–1046 BC), there was already a rhetorical method of Chinese antithesis, and both verses and prose in that period possessed extremely high antithesis. Since the Spring and Autumn Period and the Warring States Period (770–221 BC), the majority of literati had begun to use the rhetoric of confrontation. It was not until the Wei and Jin Dynasties (220–420 AD) and the Southern and Northern Dynasties (420–589 AD) that the four-six-character couplet appeared. By the time of the Tang Dynasty (618–907 AD), rhythmical prose characterized by parallelism and ornateness was quite mature [12, 13]. The couplet has existed in China for a long time; however, it never reached its peak until the Song Dynasty (960–1279 AD) and Yuan Dynasty (1271–1368 AD). In the Ming Dynasty (1368–1644 AD) and Qing Dynasty (1636–1912 AD), the couplet reached its climax. For example, “Two golden orioles sing amid the willows green; A flock of white egrets flies into the blue sky” is a very antithetical poem proposed by Du Fu (712–770 AD), a famous poet in the Tang Dynasty. Although it has developed to modern times, Chinese still has the symmetrical structure [14, 15].

### 2.2. Linguistic Topology

Topology first appeared around 1900. Linguistic topology has a special meaning that can be analyzed from three levels. The first level of meaning is that linguistic topology is transformed from psychology and then classified based on the characteristics of language pronunciation, part-of-speech, and sentence pattern [16]. It belongs to the most widely used linguistics in the field of topology. According to the above statement, every language has its own topology. Although there may be different topologies in other aspects, the function of topology is to reasonably divide languages into different categories [17, 18]. In the 19th century, some scholars focused on the lexical classification of languages in early linguistics, and this classification was regarded as the topological classification. This definition introduces the basic meaning of topology to contemporary linguistics: topology is closely connected to the comparison between different languages. Topology is a method of forming linguistic theory [19]. This means that it is a method of language analysis that establishes a linguistic theory different from other methods. In the meantime, the ideas and propositions should be closely associated with functional linguistics. Therefore, topology in this sense is often referred to as a functional topology method. The mapping between topological spaces *f*: *X−Y* is bijective, and *f* and *f−*1 are continuous; then, *f* is a homeomorphism [20]. If there is a homeomorphic mapping from *X* to *Y*, then *X* and *Y* will be considered homeomorphic, and this process is also called topological transformation. The first line of the couplet is equivalent to *X*, and the second line of the couplet is equivalent to *Y*. The first line of the couplet is entered to generate the second line of the couplet. This process is a topological mapping from the first line to the second line of the couplet [21].

### 2.3. Cognitive Linguistics

Cognitive linguistics does not belong to a particular kind of linguistics because it is also mixed with theories of cognitive neurology. Hence, it is interdisciplinary. It was born under the second generation of cognitive and empirical science based on the opposing mainstream linguistic transformational grammar, which was gradually formed from 1980 to 1990 [22, 23]. The development of cognitive linguistics has now included subjects in many fields, such as psychology, artificial intelligence, and systems theory subjects. Meanwhile, cognitive linguistics has an important theoretical basis: human beings will promote language innovation, learning, and use during the cognition process of external things since the source of human knowledge is that human beings are born with cognitive abilities. Cognitive linguistics has had a significant impact on traditional linguistics once it came into being [24].

### 2.4. Corpus Establishment

The primary concern is data; in natural language processing, it is the corpus [25]. Second, corpus must be carefully screened in terms of quality and data scale to ensure that the results generated have sufficient accuracy [26]. Due to the large capacity of computer storage, authentic text data, and fast and accurate information extraction, linguists can use the electronic corpus to describe language from multiple angles and levels, verify various language theories and hypotheses, and even establish new language models and language concept. The basic corpus adopted by the couplet system in the present work is Liweng's Guide to Rhyme (an ancient Chinese enlightenment reading material to poetry creation). Liweng's Guide to Rhyme is a corpus adopted by the Intelligent Science Laboratory for a long time. Indeed, only using Liweng's Guide to Rhyme as the corpus is far from enough, and the effect of the couplet will be not good [27, 28]. Under machine learning theories, enough language data must be gathered to ensure the automatic operation of the couplet system. At this time, a huge corpus is required, which must contain most of China's excellent couplets and poetry information for thousands of years. Therefore, before the experiment, some extra corpus is collected on the Internet to improve the quality of couplet generation. In the present work, a large number of Chinese classic couplet sentences and antithetical sentences are selected to maximize the content of the corpus used. At this stage, the construction of corpus has been completed.

### 2.5. Seq2Seq Model Establishment

#### 2.5.1. ANN

**Figure 1:**
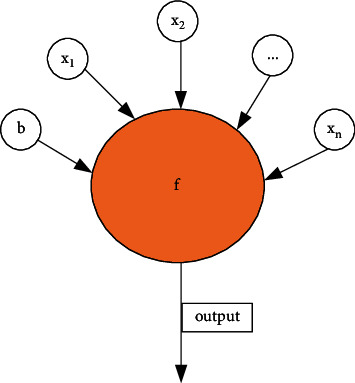
Neuron structure.

ANN is basically similar to topology, and both are cognitive models. In the present work, neural networks will constitute not only an important part of deep learning technology but also the core of the Seq2Seq couplet generation model [29]. Using the Seq2Seq model is inspired by previous literature, and it can mimic the human brain and thereby human thinking. The composition of the human brain is quite complex. According to statistics, the human brain is probably composed of nearly 100 billion neurons, and the relationship among them is intricate. Because of this complex relationship, ANN used this time also needs to build a large number of “neuron” structures, and each “neuron” is an activation function [30]. A connection is established between any two neurons, called a weighted value, which is a temporary memory of the input. The activation function will process the input accordingly. The output of the neural network is determined by the weight and the activation function. The simplest neural network consists of only one neuron [31], as shown in Figure 1.

Equation (1) describes the weighted input variables.

(1) $$\begin{array}{c} x^{j}=\sum_{i=1}^{n}x_{i}^{j-1}w_{i}^{j-1}. \end{array}$$

Equation (1) indicates that the input variable of a layer is the sum of the product of all variables of the previous layer and the weight. The activation function is an output function, which is the secondary processing of intermediate results, as shown in the following equation:

(2) $$\begin{array}{c} y_{j}=f\left(x_{j}\right). \end{array}$$

**Figure 2:**
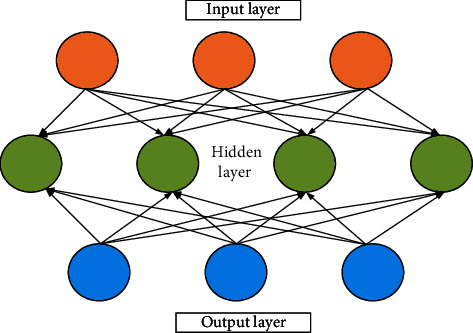
Neural network structure.

A complex neural network is composed of two interconnected neurons between two layers. Its structure is demonstrated in Figure 2.

Figure 2 displays a multi-level structure in neural networks. Its structure can be expressed by a vector as presented in the following equation:

(3) $$\begin{array}{c} Net=w^{T}x+b, \end{array}$$

where *w* represents the weight vector of each node, *x* denotes the input value of each layer, and *b* refers to the paranoid vector that usually starts from 1. The function of ANNs is clearer and more intuitive by simplifying all values to vectors.

#### 2.5.2. RNN

**Figure 3:**
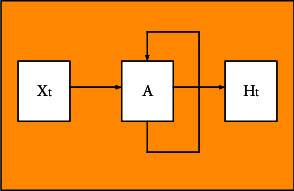
RNN structure.

ANN is the prototype of RNN. RNN is an optimization over ANN. Its structure is more complex than ANN, but it is more efficient in processing similar sequential data [32].  Figure 3 displays the simplest RNN structure.

**Figure 4:**
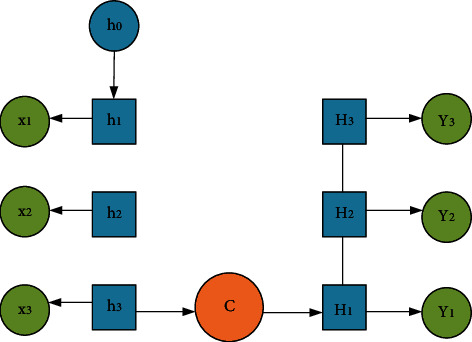
Complete RNN internal structure.

Through Figure 3, assuming a neural network is A, it is essential to obtain the input *X*_*t*_ of a certain state and then output a value *H*_*t*_. Due to cyclic use, information can be passed from the current time step to the next time step. To be specific, the same network will undergo numerous cycles in different time states, and each neuron will send the updated results to the neuron at the next moment.  Figure 4 illustrates the internal structure of an RNN.

In Figure 4, the arrow stands for the direction of signal movement towards the next step. The mathematical formulas of this process are as follows. The lateral movement can be written as

(4) $$\begin{array}{c} h_{1}=f\left(Ux_{1}+Wh_{0}+b\right). \end{array}$$

The subsequent states are the repetition of the first process, and the parameters are still *U*, *W*, and *b*. The rest can be deduced by analogy.

The equation for vertical transfer is

(5) $$\begin{array}{c} y_{1}=\text{soft}\max\left(Vh_{1}+c\right). \end{array}$$

Similarly, the subsequent output and the first output follow the same law. Each arrow is a transformation process, and on this basis, the calculation continues [33]. In the field of natural language processing, *c* is defined as a context vector for communicating encoding and decoding. Through continuous iteration, all input sequences are ultimately converted into output sequences.

Softmax function is one of the activation functions used in multi-classification problems, and it can normalize the output value to a probability value [34]. Here, it is used to optimize the output of the RNN. Assume that *C* is the number of categories to be predicted and *a* is the output; then, *C* outputs are denoted as *a*_*1*_, *a*_*2*_..., *a*_*c*_. Therefore, for a sample, the probability that it belongs to class *i* is the value of softmax function. Its mathematical expression is

(6) $$\begin{array}{c} softmax=\frac{e^{a}}{\sum_{K=1}^{C}e^{a}k}. \end{array}$$

#### 2.5.3. Seq2Seq Model

The most basic Seq2Seq model consists of three parts: the encoder, the decoder, and the intermediate state vector connecting the former two.

**Figure 5:**
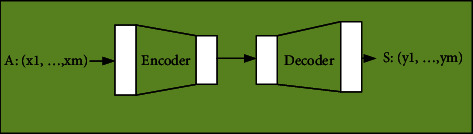
Sequence couplet automatic generation models.

First, the most basic model, the serial couplet system, is introduced. After receiving the signal of the input sentence, the sequence couplet model system performs data statistics on the special part-of-speech in the sentence by analyzing the characters in the first line of the couplet using RNN. In this way, a single vector representing the preceding clause is obtained. At this time, another RNN is used to decode the input vector into the subsequent clauses through intelligent character generation. Basically, this process generates a sequence through encoding and decoding, which is a global sentence.  Figure 5 shows the process of continuous couplet generation.

**Figure 6:**
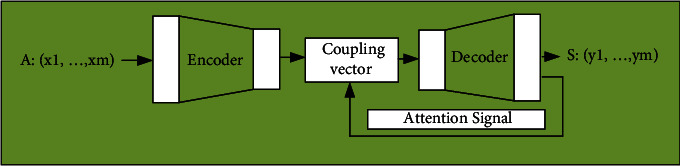
Couplet generation model under attention mechanism.

The second model is based on the couplet generation of the attention mechanism. The attention mechanism simulates the alignment between the input and output positions, which can be regarded as a local matching model. Besides, the pitch coding problem can also be solved by paired attention mechanisms.  Figure 6 displays the extension of the attention mechanism to the sequence couplet generation model.

The couplet system reported here adopts the statistical method in the field of natural language processing. Based on the principle of machine learning, the model needs continuous training. Only when the model is optimized can the performance of the couplet system improve. The purpose of model training is to optimize the parameters and algorithms, and the evaluation criteria are responsible for testing the effectiveness of the model. The loss function is such an evaluation function. Here, cross entropy is selected as a loss function. The mathematical definition of cross entropy is shown in the following equation:

(7) $$\begin{array}{c} H\left(p,q\right)=\sum_{i}p\left(i\right)log\frac{1}{q\left(i\right)}, \end{array}$$

where *p* and *q* denote two different distributions, of which one is the real distribution, and the other is the predictive distribution. The similarity between the distribution of the test set and the real situation can be calculated by (7) to judge the accuracy of the model in the test set.

### 2.6. Framework Design of Machine Learning Algorithm

**Figure 7:**
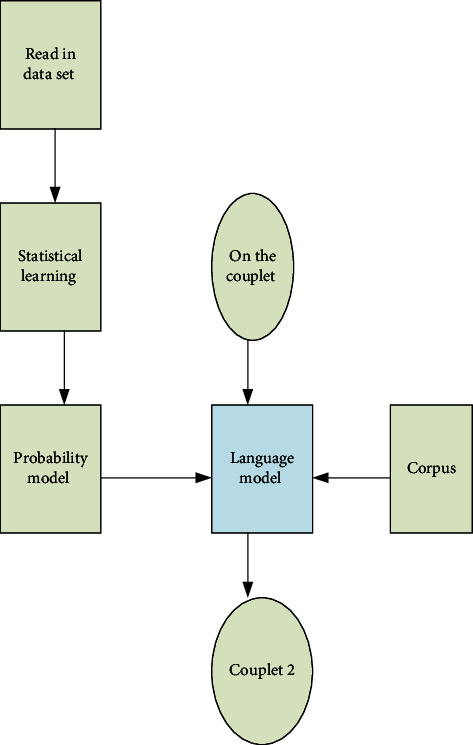
Flowchart of the couplet automatic generation system.

TensorFlow is the leader among many deep learning frameworks; thanks to its excellent performance and algorithm execution efficiency, it is preferred by many researchers and programmers [35–38]. TensorFlow has a crucial component, the client, which connects to the master and multiple workers via the session interface. Each worker is connected with other hardware devices, and simultaneously, a central processing unit or a graphics processor must be used instead to achieve the goal, thereby controlling each kind of hardware. The system flow is demonstrated in Figure 7.

Figure 7 reveals the complete development process of the algorithm. From the above flowchart, this is a typical algorithm development process including observation, experiment, model determination, test, model update, and result output, and every step is indispensable. The continuous iteration is a process of keeping improving and selecting the optimal. At the same time, the part-of-speech dimensions (16, 32, 64, and 128) of the second line of couplet output shall be tested to determine the corresponding relationship between the words in the first line and second line of couplets.

The development environment of this test is as follows:

  Hardware: a notebook computer.
  Operating system: Windows 10 system.
  Development platform: PyCharm.
  Development language: Python 3.6.
  Calculation framework: TensorFlow 1.6.

The corpus used is saved in text format. One character is stored in each line, with a total of 9,131 words.

## 3. Test Results

### 3.1. Verse Test

Test of couplet output, to a large extent, is also the test of part-of-speech accuracy. Therefore, using poem verses for the part-of-speech test can be more conducive to evaluating the rationality of the system. In the present work, the classic five-character and seven-character quatrains in Tang poems are entered in the dialog box of the first line of the couplet. Through automatic generation, the output results are very close to the first lines of the couplet and are also very neat. However, they are also quite different from the original verses, showing a unique style. Hence, the Seq2Seq model can perform targeted learning and imitation after getting the language reminder. Nevertheless, different from the couplet, poetry is not particularly demanding for antithesis. The results are summarized in Table 1.

Based on the above two examples, the output second lines do not correspond well to the first lines of the couplet, and each word does not form a sharp contrast with each other in the corresponding position. One reason is that the first line of the couplet is a verse, which itself does not require a strict antithesis.

### 3.2. Couplet Test

**Figure 8:**
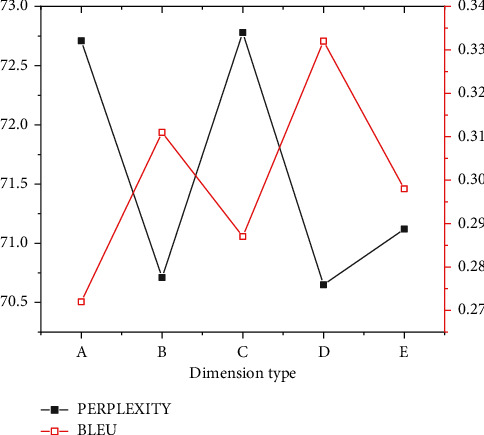
Dimension detection of part-of-speech. (a) 0 dimensions. (b) 128 dimensions. (c) 64 dimensions. (d) 32 dimensions. (e) 16 dimensions.

Regarding the ancient couplet, a verse in Liweng's Guide to Rhyme is input: “Passenger in the post encounters the plum rain.” The result given by the system is “People in the village get drunk with the wicker wind.” In contrast, the second line of the couplet in the original text is “People in the pavilion pick the lotus wind.” This example suggests that the automatically generated result does not exactly match with the first line of the couplet. Still, there is an internal connection between the part-of-speech comparison. Therefore, in the second test, the sentences in modern poems are selected. The verse “Knock the chess to find the way last night” is input as the first line of the couplet. The original text of the second line is “See my face in the mirror today.” The result of the model system is “See my smiling face in the mirror today.” Only one word is different, but the feeling is quite different; the “smiling face” is more vivid than the “face.” Therefore, the designed couplet system shows good generation results whether for the ancient couplet or the modern couplet, proving its strong adaptability.  Figure 8 presents the dimension detection of part-of-speech.

According to Figure 8, as the dimension of the part-of-speech vector becomes larger, the semantics of the word itself will change. However, regarding the score, the overall trend is stable, meaning that the first and the second lines of the couplet are better in terms of part-of-speech matching.

### 3.3. Reduplicated Word and Vocabulary Test

**Figure 9:**
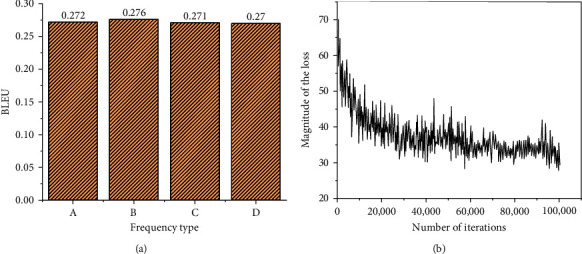
Results of the simulation experiment. (a) Performance testing of reduplicated words. (b) Result of the loss function (A: word frequency = 0; B: word frequency ≤ 1; C: word frequency ≤ 3; D: word frequency ≤ 5).

Figure 9 illustrates the results of the simulation experiment.

From the data curve in Figure 9, after a period of automatic generation training of couplets, the loss magnitude of the model gradually decreases, indicating that the generation results of the model have good accuracy. The art requirements of words in couplets are excessively high, so this experiment also tests reduplicated words. Through Figure 9, there is no obvious difference in the performance score of reduplicated words in different word frequencies, demonstrating that the model can reasonably understand and detect reduplicated words in the first line of couplets. When inputting “Fo Jiao Qing Quan Piao Piao Piao Piao Piao Xia Liang Tiao Yu Dai” in the dialog box of the first line of couplets of the system, the system outputs the second line of couplets as “Chan Xin Ming Yue Jiao Jiao Zhao Zhao Lai Lai Yi Dian Chan Xin,” failing to reach a neat confrontation. The words “Jiao Jiao Zhao Lai” in the second line of couplets are obviously wrong in part-of-speech. It is obvious that there are two groups of reduplicated words plus a verb in “Piao Piao Piao Piao Piao” in the first line of couplets, but the system only gets three groups of reduplicated words in the second line of couplets, showing no correlation. Therefore, this system cannot precisely recognize reduplicated words in the first line of couplets for verse, and the accuracy of the algorithm needs to be improved. There are also two other groups of experiments to detect reduplicated words, and the two pairs of couplets are “Shan Shan Shui Shui Chu Chu Ming Ming Xiu Xiu, Jia Jia Hu Hu Jia Jia Hao Hao Qi Qi” and “Xiu Xiu Ling Ling Yuan Yuan Jin Jin Cui Cui, Chun Zhen Pu Shi Chun Du Chun Pu Hou Hou Chun Zhen,” respectively. Through the above three pairs of couplets, evidently, when dealing with a single word, the response of the system is often not satisfactory. The second line of couplets automatically generated by the system has low matching degree and relevance with the second line of couplets, and the output results are just a set of words similar in form but without particular meaning.

### 3.4. Boundary Test

According to actual application requirements, the maximum boundary value of the software presentation box must be checked. What is applied is a unique method in software engineering: black box testing. The test results are summarized in Table 2.

In the initial stage of the system, there is no input for the first line of the couplet, and the word count at this time is judged to be zero. If the first line of the couplet enters excessive words in the dialog box, the second line of the couplet will issue an early warning signal during the generation process, reminding “Too many words entered, please try again.” This form of warning is similar to the reminder when the password does not match on the usual login page. If no word is entered in the first line of the couplet, no result will be generated in the second line of the couplet. When the number of words entered by the user is greater than 50 words, the system cannot continue. Because the number of words is strictly controlled when setting the system program: the above result will appear when the number of words input is greater than 50 words. Under normal circumstances, the number of the first line of the couplet input by the user will not exceed 30 words. When it exceeds 50 words, the couplet itself does not have any practical application value.

## 4. Discussion

Through experiments, the Seq2Seq model can handle the first line of the couplet in different categories reasonably, including the response to punctuation. In the meantime, the first and the second lines of the couplet present high connection and neatness. However, after many times of debugging, some weaknesses of the model are discovered. When the Seq2Seq model deals with single words, the words generated cannot be accurately matched with the words of the first line of the couplet. Only when the number of input words is large can the automatically generated second line of the couplet be improved.

In future works, improvement over corpus should be the first concern. Corpus is the most basic and most vital link to the entire system. If it cannot provide sufficient language content or has excessive errors, inaccurate results or high error rates will occur. In the entire couplet automatic generation system, the selection of corpus must not only have a strong pertinence but also allow the corpus to possess the generalization capability. Second, in terms of language model, couplet itself has many characteristics (the flatness of grammar, the correspondence of part-of-speech, and the degree of semantic relevance). These two aspects are not considered in the present work while establishing the language model. In this case, the next step is to optimize these problems. For example, at this stage, other algorithms can be added to the original model, and some words, phrases, and sentences that cannot meet the requirements can be deleted before the language model can be reestablished and executed. In the end, the designed model can be further optimized regarding “matching degree” and “language expression habits” while meeting performance requirements. According to the test of the automatic couplet system launched by Microsoft Research Asia, many output results are very unsatisfactory. Meanwhile, as artificial intelligence develops quickly, various algorithms can be optimized continuously.

## 5. Conclusion

Under cognitive linguistics, the present work aims to automatically generate couplets using the linguistic topology of DNN and analyze the DNN outputs.

The symmetry, topology, and cognitive linguistics of language are explored to lay a theoretical foundation; next, RNN is employed to build the Seq2Seq model, which is then implemented automatically on TensorFlow. Results demonstrate that after the corpus is input, the output five-character quatrain, seven-character quatrain, part-of-speech, and couplet all present good correspondence. The couplets can be generated quickly and meet the requirements on rhyme and couplet matching. Still, this model has some limitations. The corpus used is not rich enough. Only one corpus is input, far from satisfying the requirements on couplet richness. Besides, the confrontation of couplet is not only the correspondence between words but also the association between phrases. Moreover, the requirements on words in modern couplets are higher, so that further optimization of the model system is required. Hopefully, the model can promote traditional Chinese culture by integrating artificial intelligence and other advanced technologies, in an effort to achieve better cultural inheritance and absorption.

## Figures and Tables

**Table 1:** Output results.

Topology	Input	Output
Five-character quatrain	Lush, lush grass on the plain	Red, red flower in the water
Seven-character quatrain	The morn rain o' Wei Town has laid the light dust clean	The spring wind o' Qinling Mountain has dyed the green lake blue

**Table 2:** Statistics on the output categories.

Input topology	Output topology
0 words	No output
0–50 words	Normal output
≥50 words	Too many words entered, please try again.

## Data Availability

The raw data supporting the conclusions of this article will be made available by the authors, without undue reservation.
